# Pneumocystis pneumonia in a patient with diabetes mellitus: A case report

**DOI:** 10.1097/MD.0000000000032290

**Published:** 2023-02-03

**Authors:** Ziling Li, Shuyun Xu, Jing Shi, Yong Zhang

**Affiliations:** a Department of Respiratory and Critical Care Medicine, Tongji Hospital, Tongji Medical College, Huazhong University of Science and Technology, Wuhan, Hubei Province, China; b Department of Pediatrics, Tongji Hospital, Tongji Medical College, Huazhong University of Science and Technology, Wuhan, Hubei Province, China.

**Keywords:** diabetes mellitus, *Pneumocystis jirovecii*, *Pneumocystis pneumonia*

## Abstract

Pneumocystis pneumonia (PCP) is an opportunistic infection of patients with congenital or acquired immunodeficiency. It is most frequently occurred in human immunodeficiency virus (HIV) infection, organ transplantation, leukemia, and immunosuppressive therapy. Here we describe the rare case of PCP in a non-HIV-infected diabetic patient and find possible reasons for the association through a literature review.

**Patient concerns:**

A 65-years-old male was admitted to our hospital due to a 10-year history of abnormal blood glucose levels and edema of both lower extremities for half a month. However, the patient developed a high fever and progressive dyspnea during hospitalization.

**Diagnoses:**

The patient had elevated blood sugar levels, a low white blood cell count within normal limits, and severe lymphopenia. His blood *G* test and lactate dehydrogenase levels increased significantly. Multiple sputa and bronchoalveolar lavage fluid specimens for *Pneumocystis jirovecii* (PJ) nucleic acid detection were positive. Chest computed tomography scan demonstrated hazy patchy shadows in the lungs suspected to be pulmonary infections. No tumor, transplantation, or an autoimmune disease was found in the examinations. The patient was diagnosed with PCP finally.

**Interventions:**

A combination of oral trimethoprim-sulfamethoxazole and intravenous caspofungin was administered immediately against PJ. The patient was also treated with noninvasive ventilator-assisted ventilation, subcutaneous insulin, and hemodialysis therapy.

**Outcomes:**

The patient was discharged home finally with a fair general condition and was followed up without respiratory symptoms.

**Lessons:**

The compromised immunity in HIV-negative patients with diabetes may be related to lymphocyte decrease and dysfunction, which may cause diabetic patients prone to PJ. Although PCP is rare in diabetes, it should be paid attention to the high rate of misdiagnosis and missed diagnosis.

## 1. Introduction

*Pneumocystis pneumonia* (PCP) is a lethal opportunistic infection caused by *Pneumocystis jirovecii* (PJ) in immunocompromised patients.^[1,2]^ It is most prevalent in human immunodeficiency virus (HIV)-infected patients, especially those with CD4^ + ^*T* cell counts less than 200 cells/*µ*L, whereas *T* lineage lymphopenia and dysfunction increase susceptibility to PJ infections significantly.^[3]^ However, the immune function is compromised in diabetic patients, which may be associated with abnormal *T* lymphocyte function.^[4,5]^ Only a few reports of PCP have focused on non-HIV-infected diabetic patients, and the association between diabetes and PCP is unknown.^[6,7]^ In contrast to HIV-positive patients, PCP in HIV-negative patients has a more abrupt onset, more rapid progress, and a poor prognosis with a mortality rate of 30.6%.^[2,8]^ We herein report a rare case of PCP in diabetes mellitus, aiming to enhance clinical awareness, improve outcomes, and avoid misdiagnosis or missed diagnosis.

## 2. Case report

A 65-years-old male with a 10-year history of abnormal blood glucose levels and edema of both lower extremities for half a month, was admitted to our nephrology department on September 20, 2022. Ten years ago, the patient was diagnosed with type 2 diabetes mellitus during a medical checkup and treated with unspecified oral hypoglycemic agents without routine blood glucose monitoring. Approximately 4 years ago, the patient was diagnosed of having diabetic nephropathy with elevated blood creatinine (Cr) levels, which was poorly controlled with oral medications such as compound *α*-ketoacid tablets, beraprost sodium, and medicinal charcoal tablets. Generalized weakness and bilateral lower limbs edema occurred 15 days previously without obvious inducement, accompanied by decreased appetite and abdominal distention. There was no fever, cough, expectoration, backache, dysuria, urinary frequency, urgency, or any other discomfort. The blood urea nitrogen was 31.83 mmol/L (normal reference ranges: 3.6–9.5 mmol/L) and Cr 403.8 *µ*mol/L (59–104 *µ*mol/L) on September 13, 2022. Following symptomatic therapy at a local hospital, his symptoms alleviated mildly. The patient presented to our nephrology outpatient clinic for further treatments and was admitted with the diagnosis of type 2 diabetes complicated by end-stage renal disease. The patient had been hypertensive for 3 years, and the blood pressure was well controlled by taking nifedipine controlled release tablets 30 mg twice a day. He denied smoking, previous surgery, or any family history. Physical examination on admission revealed a temperature of 36.5°C, a heart rate of 104 bpm, a respiratory rate of 20, and a blood pressure of 101/56 mmHg. Both lungs had clear respiratory sounds and no rales. Auxiliary examinations were performed: white blood cell (WBC) 5.90 × 10^9^/L (3.50–9.50 × 10^9^/L), neutrophils (N) 4.90 × 10^9^/L (1.80–6.30 × 10^9^/L), lymphocytes 0.48 × 10^9^/L (1.10–3.20 × 10^9^/L), hemoglobin 57 g/L (130–175 g/L); albumin 32.5 g/L (35–52 g/L), globulin 23.9 g/L (20–35 g/L); hepatitis B virus, hepatitis C virus, HIV and Treponema pallidum tests were negative; rheumatism immunity series, anti-neutrophil cytoplasmic antibodies and anti-glomerular basement membranes antibodies were negative; urine protein (++); urine erythrocytes (-), urine leukocytes (-), urinary casts (-); 24-hour urine total trace protein: 2043.2 mg/24 hours (≤140 mg/24 hours); 24-hour urine trace albumin: 1328.8 mg/24 hours (≤30 mg/24 hours); a routine fecal test was normal. An ultrasonic cardiogram revealed the enlargement of the left atrium and the widening of the proximal ascending aorta. Renal ultrasonography showed small kidneys with increased parenchymal echogenicity bilaterally. The levels of glycated hemoglobin were 7.6% (4.0%–6.0%); thus, subcutaneous insulin glargine and insulin aspart were administered to control his blood glucose levels. According to the patient’s blood glucose levels, insulin dosages were modified.

**Figure 1.**
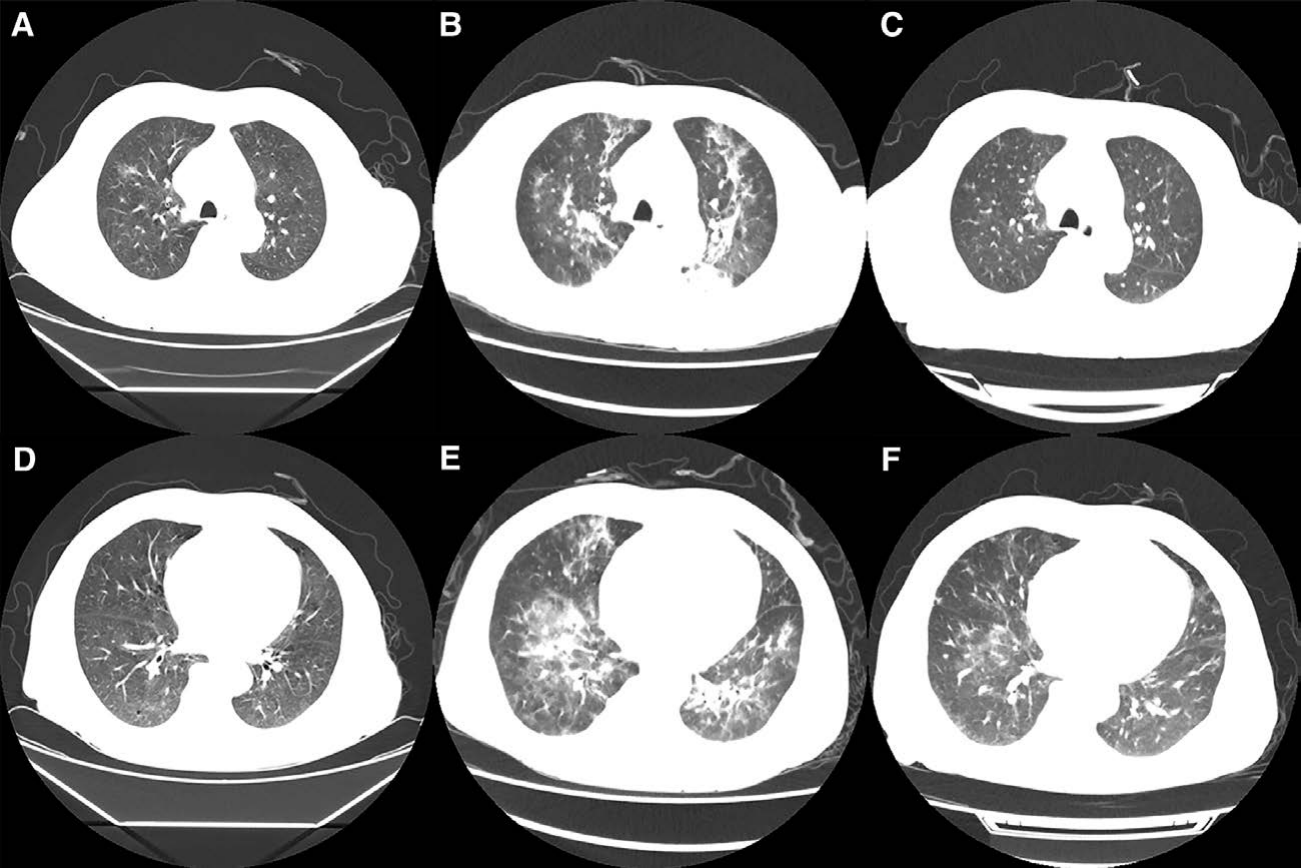
Chest CT scans of the patient. (A, D) Pulmonary window of lung CT demonstrated patchy shadows on September 21, 2022; (B, E) Lung CT scan on September 28 showed progressive infections; (C, F) CT scan revealed the regression of the changes in the lungs after treatments on October 9, 2022. Upper panel (A–C): imaging manifestations of of both upper lobes. Lower panel (D–F): imaging findings of the bilateral lower lobes of lungs. CT = computed tomography.

After admission, the patient began to develop a fever that peaked at 39.4°C without obvious inducement, accompanied by a cough and a small amount of white, viscous sputum. Respiratory examination showed coarse breath sounds without any rales. Laboratory examinations: WBC 5.31 × 10^9^/L, N 4.62 × 10^9^/L, L 0.27 × 10^9^/L, hemoglobin 66 g/L, erythrocyte sedimentation rate 123 mm/hours (0–15 mm/hours); hypersensitive *C*-reactive protein 94.6 mg/L; procalcitonin 0.42 ng/mL (0–0.05 ng/mL). Infectious examinations including respiratory syncytial virus, adenovirus, coxsackievirus B, cytomegalovirus, rubella virus, herpes simplex virus, *Mycoplasma pneumoniae*, and *Chlamydia pneumoniae* IgM levels were all negative. Blood nucleic acid detections of Epstein-Barr virus and cytomegalovirus were negative, too. Furthermore, the sputum smear samples for acid-fast bacilli were negative. Multiple sputum cultures and blood cultures showed negative results. A chest computed tomography (CT) scan done on September 21, 2022, demonstrated hazy patchy shadows in the upper lobe of the right lung and bilateral lower lobes, suggesting pulmonary infection (Fig. 1A, 1D). After empiric treatment with intravenous levofloxacin, moxifloxacin, and meropenem, combined with ganciclovir sequentially, the patient still had a fever. The symptoms of dyspnea appeared and worsened progressively. A sputum specimen of PJ nucleic acid detection was submitted on September 23, 2022. And the result was released to be positive on September 25, 2022. His oxygen saturation fluctuated between 85% to 88%, with 10 L of oxygen therapy delivered through a mask on September 26, 2022. The patient was transferred to the respiratory intensive care unit (RICU) on the same day.

Following admission to the RICU, the patient’s blood oxygen saturation rose to 98%, gradually assisted by a noninvasive respirator with 100% inspiratory oxygen concentration. Meanwhile, the anti-infection therapy was changed to the combination of oral trimethoprim-sulfamethoxazole (TMP-SMZ) (0.96 g/day), intravenous caspofungin (50 mg/day) plus piperacillin-tazobactam (2.5 g/day). More examinations were conducted: serum (1, 3)-*β*-D glucan (*G* test) was 429.01 pg/mL (<70 pg/mL); serum galactomannan (GM test) was negative; lactate dehydrogenase (LDH) was 453 U/L (135–225 U/L); PJ nucleic acid test of sputum samples was positive again. The diagnosis of PCP was thus clarified. The patient’s body temperature returned to normal on September 28, 2022, indicating the effectiveness of the treatment. His dyspnea also eased markedly, and the blood oxygen saturation was maintained at 100% assisted by a noninvasive respirator with 40% inspiratory oxygen concentration. The chest CT scan was re-checked on September 28, 2022, which showed increased texture in both lungs, multiple irregular patchy or striped shadows, and a small quantity of bilateral pleural effusion (Fig. 1B, 1E). Together with the patient’s history and symptoms, PCP with pulmonary edema was considered. A bedside bronchoscopy was performed on September 29, 2022, and small amounts of white secretion were observed. Both nucleic acid testing for PJ and metagenomics next-generation sequencing revealed the presence of PJ in bronchoalveolar lavage fluid from the upper and middle lobes of the right lung. These positive results were consistent with previous sputum samples. Because the level of his serum Cr rose to 609 *µ*mol/L and *N*-terminal pro-*B*-type natriuretic peptide was 5818 pg/mL (<241 pg/mL), the patient was considered to have PCP with pulmonary edema clinically, and hence, he began hemodialysis therapy on September 27, 2022. Afterward, the patient’s condition improved steadily, with an oxygen saturation of 96% to 98% on 3 L/minutes of oxygen through a nasal cannula. The patient was shifted from RICU to the general ward at the department of nephrology on September 30, 2022. He has been prescribed a combination of oral TMP-SMZ (0.96 g/day) and intravenous caspofungin (50 mg/day). Arteriovenous fistula angioplasty for hemodialysis was performed later. A re-check blood investigation on October 8 showed that the *G* test decreased to 275.24 pg/mL and LDH decreased to 240 U/L. The third chest CT examination on October 9 indicated the significantly alleviated lung infection and absorption of bilateral pleural effusions (Fig. 1C, 1F). Routine blood re-examination reported that WBC was 7.82 × 10^9^/L, N was 5.21 × 10^9^/L, and L was 1.93 × 10^9^/L on October 11, 2022. After anti-infective and other symptomatic support treatments, the patient’s condition improved considerably and he was discharged home finally on October 18, 2022. The general condition of the patient was fair, with no fever. The patient continued to take TMP-SMZ orally to fight PJ infection, inject insulin subcutaneously to control blood glucose levels, and underwent regular hemodialysis treatment after discharge. A follow-up presented that the patient had no febrile, cough, chest tightness, dyspnea, or other symptoms.

## 3. Discussion

Genetic studies have demonstrated that *Pneumocystis* belongs to the yeast class of fungal organisms rather than protozoan parasites.^[9]^ *Pneumocystis carinii* infects mice, whereas PJ infects humans via aerosol transmission.^[1]^ PJ exhibited a strong affinity for alveolar epithelial cells that can lead to severe pneumonia. Its capsule is rich in *β*-D-glucan, which can stimulate pulmonary inflammation and is a target for echinocandins.^[10,11]^ Many HIV-negative immunocompetent individuals are susceptible to PJ infections apart from HIV-positive patients, including those with malignancy, immunosuppressive therapy, organ transplantation, and preterm neonates.^[12–14]^ The incidence rate of PCP in HIV-negative patients is increasing year by year. Their condition often progresses rapidly, and they are at high risk for respiratory failure and death.^[15]^

Patients with PCP typically exhibit severe symptoms and mild signs, and the primary clinical manifestations include progressive dyspnea, fever, dry cough, and other respiratory symptoms.^[11]^ In addition to elevated blood *G* tests, serum LDH levels in PCP patients are markedly elevated, which may indicate underlying inflammation and injury in the lungs, as well as the severity of the disease.^[16,17]^ The patient had a rapid onset and rapid progression, requiring noninvasive ventilator-assisted ventilation; the patient had typical clinical symptoms and signs of PCP. During the course of the disease, the patient had positive PJ nucleic acid detection in sputum and bronchoalveolar lavage fluid many times. His blood G test and LDH significantly increased, and chest CT showed multiple fuzzy patches in both lungs. All of these were highly consistent with the characteristics of PCP. Furthermore, no evidence of pathogens other than PJ was found, so the diagnosis of severe PCP in this patient was clear.

This patient is an HIV-negative elderly male who has never received glucocorticoid treatment and has a 10-year history of diabetes. No common PCP susceptibility factors such as a tumor, transplantation, or autoimmune disease were found in the complete series of examinations. The PCP case report is considered to be related to diabetes. A few reports have studied diabetic patients with PCP, but neither clarified the association between PCP and diabetes at the time.^[6,7]^

Diabetic patients with poor glycemic control are more likely to develop PCP, which may be associated with a compromised immune system because of hyperglycemia.^[7]^ It is the leading cause of compromised immune that a series of metabolic changes can be triggered by hyperglycemia, such as suppression of neutrophils and mononuclear phagocyte systems, as well as lymphocyte dysfunction, inhibiting the lungs’ ability to eliminate pathogens.^[18]^ Studies have demonstrated that diabetes can reduce the activity of NK cells, alter *T* cell differentiation, and impair the function of *B* cells.^[4]^ Moreover, researchers have found fewer CD4^ + ^*T* cells or CD8^ + ^*T* cells in diabetic patients.^[5,19]^ Severe lymphopenia is a significant risk factor for PCP in an immunocompromised population.^[20]^ It has been shown above that immune compromised diabetic patients are more prone to PJ. The patient in this case has elevated blood sugar levels, a low white blood cell count within normal limits, and a significant decrease in lymphocytes. The aforementioned evidence suggests that patients with long-term poor blood sugar control become immunocompromised, which may be the cause of PCP.

Currently, TMP-SMZ is the first-line treatment for PCP, and its combination with caspofungin can significantly reduce mortality in severe PCP patients.^[21,22]^ As soon as PCP was suspected, this patient was treated with TMP-SMZ and caspofungin while kidney function was closely monitoring kidney function. We adjusted the dosages based on his kidney function and supplemented them with sodium bicarbonate tablets. Overall, diabetic patients may have PCP, so attention should be paid to avoid misdiagnosis or missed diagnosis in the clinic.

## Acknowledgments

The study was supported by the Teaching Research Project of the Second Clinical College, Huazhong University of Science and Technology (Grant No. 202029).

## Author contributions

**Conceptualization:** Shuyun Xu, Jing Shi, Yong Zhang.

**Data curation:** Ziling Li, Shuyun Xu.

**Investigation:** Ziling Li, Yong Zhang.

**Writing - original draft:** Ziling Li, Jing Shi.

**Writing - review & editing:** Shuyun Xu, Jing Shi, Yong Zhang.
